# Association between polyunsaturated fatty acid intake and the prevalence of erectile dysfunction: A cross-sectional analysis of the NHANES 2001–2004

**DOI:** 10.1186/s12944-023-01950-9

**Published:** 2023-10-25

**Authors:** Yong Huang, Yingying Wang, Huiyi Su, Hexi Wang, Haoyu Xu, Chengwei Xu, Fulin Zhou, Yao Zhang

**Affiliations:** 1 Department of Urology, The First Affiliated Hospital of Chongqing Medical University, Chongqing, 400016 China; 2 Department of Oncology, People Hospital of Jiulongpo District, Chongqing, 400050 China; 3 Department of Respiratory Medicine, Children’s Hospital of Chongqing Medical University, Chongqing, 400014 China

**Keywords:** Polyunsaturated fatty acids (PUFAs), Arachidonic acid (AA), Erectile dysfunction (ED), National Health and Nutrition Examination Survey (NHANES)

## Abstract

**Background:**

Polyunsaturated fatty acids (PUFAs) have demonstrated significant therapeutic potential across a wide range of disease. The aim of this study was to investigate the potential impact of PUFA intake on the prevalence of erectile dysfunction (ED).

**Methods:**

The study included a total of 3730 participants from the National Health and Nutrition Examination Survey (NHANES) 2001–2004. Univariate analysis, multivariate regression analysis, subgroup analysis and machine learning were utilized to explore the relationship of variables to ED. Dose response curves were constructed to observe the linear or nonlinear relationship between PUFA intake and the prevalence of ED. Propensity score matching (PSM) was used for sensitivity analysis. Finally, the potential mechanistic link between PUFA intake and ED was explored.

**Results:**

Through univariate and multivariate regression analysis results before and after PSM and XGBoost algorithm model results, arachidonic acid (AA) was chosen as the main research object. The consumption of AA was found to be associated with a decreased prevalence of ED under the fully adjusted model [OR = 0.33 (0.20, 0.56), *P* < 0.001]. The interaction between AA and education was found in the subgroup analysis. Dose-response curves indicated a linear negative correlation between AA intake and the prevalence of ED. The above results were confirmed in the data analysis after 1:1 PSM. In addition, AA intake was associated with a decrease in inflammatory biomarkers and homocysteine.

**Conclusions:**

The results suggest that AA intake is negatively correlated with the prevalence of ED. Further, anti-inflammatory and anti-endothelial damage may play a role in this.

**Supplementary Information:**

The online version contains supplementary material available at 10.1186/s12944-023-01950-9.

## Introduction

Inability to have satisfactory sexual behavior as the penis cannot achieve or maintain an adequate erection is defined as erectile dysfunction (ED) [1]. As a result of ED, patients and their partners can experience significant difficulties with their quality of life [2]. The Massachusetts Male Aging Study, conducted among individuals aged 40 to 70 years, revealed that mild or moderate ED was prevalent in 52% of the population, while the prevalence of complete ED increased from 5 to 15% with advancing age [3]. A survey of the epidemiology of ED in people aged 40 to 70 in four countries also indicated a prevalence of 9–54% with increasing age [4]. The incidence of ED exceeds 40% in American males over the age of 40. In addition, it is anticipated that ED will impact approximately 322 million men across the globe by 2025 [5].

The aetiology of ED is multifactorial, and it is currently considered to be a social-psychological-physiological disease [6]. The risk factors associated with ED are numerous, including age, cardiovascular disease, hypertension, body mass index (BMI), diabetes, lack of exercise, hyperhomocysteinemia and smoking [7–10]. Inflammation and endothelial dysfunction are important factors that promote ED [11, 12]. Inflammation not only affects endothelial cells but also contributes to the pathogenesis of metabolic syndromes, all of which promote ED [9]. Chunhui Liu et al. found that the prevalence of ED increased with increasing inflammatory indices [13]. A study by Youssef MK Farag showed that vitamin D deficiency was independently associated with a higher prevalence of ED and that vitamin D may improve ED through improving inflammation and endothelial function [14]. Therefore, the use of modifiable factors such as diet to reduce inflammation and protect endothelial function to prevent ED has attracted widespread attention.

Polyunsaturated fatty acids (PUFAs) have been proven to have beneficial effects in many diseases such as osteoarthritis, metabolic syndrome, cardiovascular disease, and bipolar disorder, through their anti-inflammatory and reducing oxidative stress effects [15–17]. ω-6 PUFAs, such as arachidonic acid (AA), are precursors of bioactive lipids. When stimulated by inflammation, AA promotes the production of leukotrienes (LTs), prostaglandins (PGs) and thromboxanes (TXs), which promote inflammation [18]. However, studies have shown that metabolites derived from AA are also involved in the breakdown of inflammation [19]. In addition, PUFAs have been shown to have a protective effect against ED by regulating key enzymes involved in ED [20]. However, a study in a rodent model suggested that a diet rich in PUFAs may promote the progression of ED [21].

The relationship between PUFAs and ED remains contradictory. Therefore, the aim of this study was to explore whether the intake of PUFAs is related to the prevalence of ED and to further explore the mechanism. The present findings can provide the basis for preventing ED in terms of dietary interventions.

## Methods

### Data source

The NHANES is a program in the U.S. that contains a wealth of nutrition and health information for children and adults in the country. Starting in 1999, a complex sampling design was used to survey approximately 5000 participants annually across the country. Then, data were collected on the sociodemographic status, physical examination parameters, laboratory test indicators, diet and questionnaire information. The program has been continually updated, with the latest survey data released every two years for open access by researchers. All participants provided informed consent forms. Detailed information about the program is available on the website (https://www.cdc.gov/nchs/nhanes).

### Study population

**Fig. 1:**
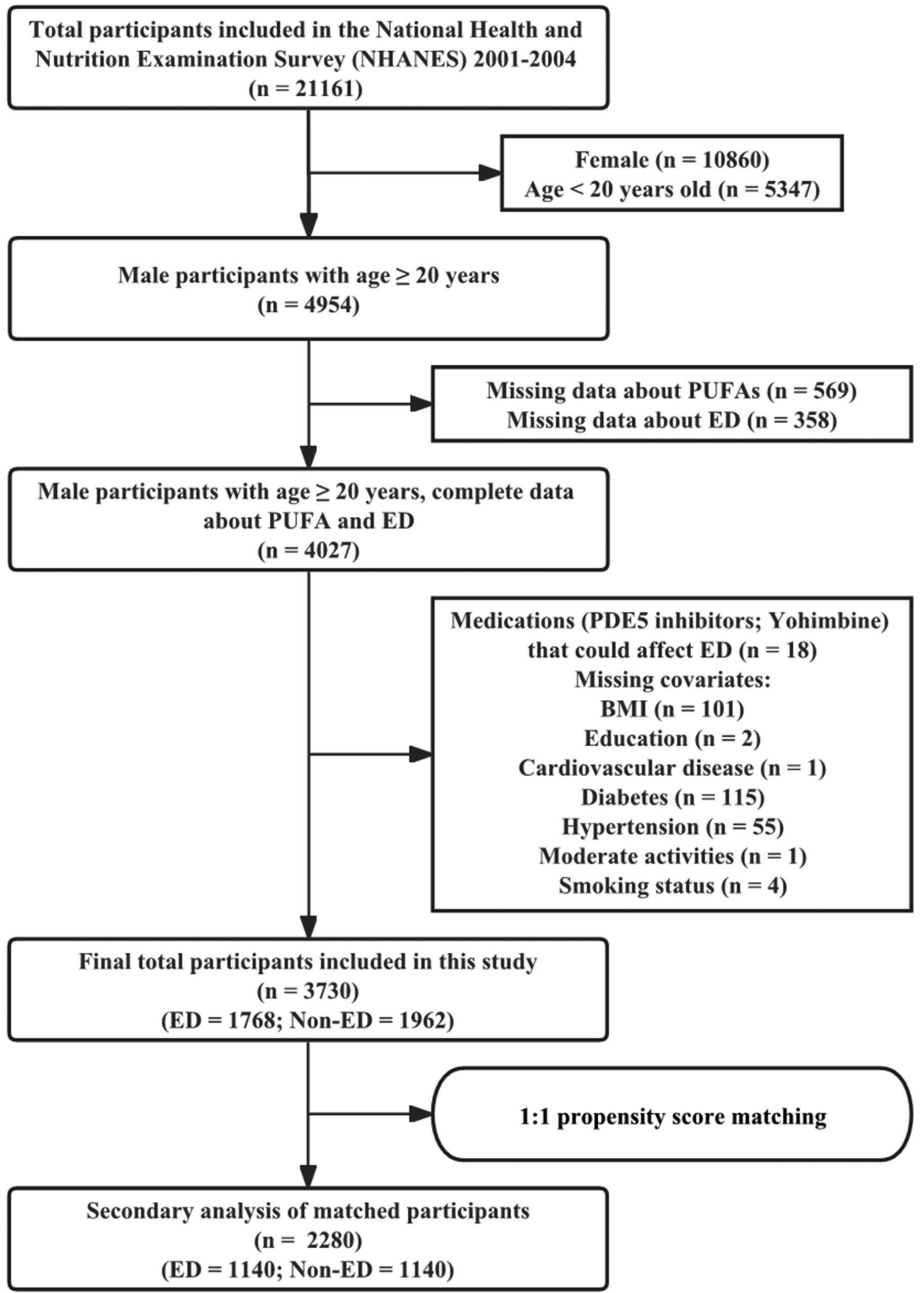
Screening flow chart of the study population. **Abbreviations**: PUFAs, polyunsaturated fatty acids; ED, erectile dysfunction; BMI, body mass index

Two cycles of NHANES data (2001–2004) were used for analysis in this study. This time range was of interest to us because the information utilized to assess ED was only investigated during this period. A total of 21,161 participants were surveyed from 2001 to 2004. The study population was excluded based on the following criteria: (1) female (N = 10,860); (2) age < 20 years (N = 5347); (3) missing data about PUFAs (n = 569); (4) missing data about ED (n = 358); (5) medications (e.g., PDE5 inhibitors, yohimbine) that could affect ED (n = 18); and (6) missing data about covariates (n = 279). Finally, through the above screening procedures, the study included a total of 3730 participants, including 1768 with ED and 1962 without ED. The detailed screening process is shown in Fig. 1.

### Measurement of PUFAs

24-hour dietary recall interviews were used to obtain dietary data. In the NHANES, the method modified by Lagerstedt et al. was used to determine dietary fatty acid content [22]. Thus, the study included seven different PUFA intakes for each participant. Specifically, docosahexaenoic acid (DHA), eicosapentaenoic acid (EPA), α-linolenic acid (ALA), arachidonic acid (AA), octadecanotetraenoic acid (SDA), docosapentaenoic acid (DPA) and linoleic acid (LA). Then, the study calculated the intake of ω-3 PUFA and ω-6 PUFA; ω-3 PUFA includes ALA (g), SDA (g), DPA (g), EPA (g) and DHA (g), and ω-6 PUFA includes LA (g) and AA (g). Eventually, the ω-3 PUFA and ω -6 PUFA intake values were added together as the total polyunsaturated fatty acid (TPFA) intake.

### Measurement of ED

ED was assessed by answering the following question: “How would you describe your ability to achieve and maintain sufficient erections for satisfactory intercourse?” This query had been proven to accurately identify men with a clinical diagnosis of ED [23]. Participants were given four options, “almost always or always able”, “usually able”, “sometimes able” and “never able”. Unlike previous definitions of ED through answering the above question as “never able” or “sometimes able” or answering “yes” to the question “Have you ever had ED?” [13, 24], herein, a stricter definition of ED was used: Participants who answered “almost always or always able” were identified as having no ED, and the rest were identified as having ED [14, 25].

### Measurement of covariates

Based on previously published articles, the study selected the following potential confounding factors that could affect ED results [7, 26–29]. These covariates were related to demographic data, physical examination, personal life circumstances, and disease history. The only continuous variable was BMI. Categorical variables included age, race, education, hypertension, cardiovascular disease, diabetes, vigorous activity, moderate activity and smoking status. Diastolic pressure ≥ 90 mmHg or systolic blood pressure ≥ 140 mmHg or been taking medications for high blood pressure was defined as hypertension. Cardiovascular diseases included stroke, congestive heart failure, myocardial infarction, angina and coronary heart disease. Diabetes was considered if glycosylated hemoglobin was ≥ 6.5% or fasting blood glucose was ≥ 126 mg/dl. Vigorous (/moderate) activity was defined as having done at least 10 min of vigorous (/moderate) exercise in the past 30 days. By answering questions “In your entire life, have you smoked at least 100 cigarettes” and “Do you now smoke cigarettes”, participants were classified into current smoker, previous smoker and non smoker.

### Measurement of possible mediating factors

Previously published articles have demonstrated the role of inflammation and homocysteine in promoting ED [30, 31]. Therefore, the following inflammatory biomarkers that may be associated with ED were selected in this study, such as white blood cell (WBC, 1000 cells/μl), lymphocytes (L, 1000 cells/μl); neutrophils (N, 1000 cells/μl), neutrophil-to-HDL cholesterol ratio (NHR), neutrophil-to-lymphocyte ratio (NLR), monocytes (M, 1000 cells/μl), monocyte-to-HDL cholesterol ratio (MHR), C-reactive protein (CRP, mg/l), CRP-to-lymphocyte ratio (CLR), CRP-to-albumin ratio (CAR) and alkaline phosphatase (ALP, U/l) [32–36].

### Statistical analysis

To ensure that the study population was nationally representative, the sample weights were used in the process of data analysis provided by NHANES. After classifying participants according to ED, baseline characteristics were described. Specifically, the percentage and mean ± standard deviation (SD) were utilized to express categorical and continuous variables, and the *P*-value was calculated by a weighted chi-square test and weighted linear regression model, respectively. Before analyzing the relationship between PUFAs and ED, covariates were checked and screened, and covariates with a variance inflation factor (VIF) ≥ 5 were eliminated. In addition, the relationships between the variables and ED were observed through univariate analysis. Next, the study constructed three adjustment models to explore the associations between PUFAs and ED in multivariate regression analysis. Model 1 did not adjust for any covariates as a crude model. Age, BMI, race and education were adjusted in Model 2. In Model 3, hypertension, cardiovascular disease, diabetes, vigorous activity, moderate activity, and smoking status were added on the basis of Model 2. Furthermore, PUFAs were divided into four groups (Quartile grouping : Q1, Q2, Q3 and Q4), with Q1 as the reference value, and a trend test was conducted. Then, the stratified correlation between PUFAs and ED was then explored in subgroup analysis.

In addition, the XGBoost algorithm model in machine learning was used to predict the relative importance of the effects of various variables on ED. Afterward, the dose response relationship between PUFAs and ED was demonstrated through the construction of restricted cubic splines (RCS) and smooth curve fitting. In the RCS, the study set the knot as four and took the value of PUFAs when OR = 1 as the reference value to explore whether there was a nonlinear relationship between the independent variable (e.g., AA) and the outcome (e.g., ED). Under the fully adjusted model (Model 3), a generalized additive model (GAM) was constructed for smooth curve fitting to verify the dose response relationship.

1:1 propensity score matching (PSM) was performed using R Software’s “MatchIt” package to balance out covariate differences between ED and non-ED groups. Matching propensity scores were performed for age, race, education, BMI, hypertension, cardiovascular disease, diabetes, vigorous activity, moderate activity, and smoking status to ensure that the ED and non-ED groups had similar covariate distributions^[13]^. Then, the study population after PSM was analyzed again to further test the validity of the results. In the above multivariate regression analysis, PUFAs with robust results were selected for subsequent analysis by comparing the results before and after PSM.

In addition, this study explored the association between PUFAs and inflammatory biomarkers and homocysteine. On this basis, the potential mechanism between PUFAs and ED was explored.

Empowerstats Software (Version 2.0) and R Software (Version 4.2.1) were used for all statistical analysis processes. In this study, the statistically significant criterion was *P* < 0.05.

## Results

### Study population basic characteristics

The weighted basic characteristics of the study participants are shown in Table 1. The study found that participants with ED were predominantly ≥ 40 years in age. Compared with people without ED, they had a higher BMI; lower education attainment; less vigorous or moderate activity; a history of hypertension, cardiovascular disease, and diabetes; and greater likelihood of smoking. More importantly, participants with ED had a lower intake of PUFAs, including TPFA, ω-3 PUFA and ω-6 PUFA, especially ALA, LA and AA.

**Table 1:** The weighted basic characteristics of the study population before PSM

Characteristics	Total	Erectile dysfunction		*P*-value
		No	Yes	
Total participants	3730	1962	1768	
Age (%)				< 0.0001
< 40 years	39.76	51.65	19.72	
≥ 40 years	60.24	48.35	80.28	
BMI (kg/m^2^), Mean ± SD	28.08 ± 5.44	27.69 ± 5.13	28.74 ± 5.87	< 0.0001
TPFA (g), Mean ± SD	20.257 ± 12.840	20.902 ± 12.594	19.170 ± 13.173	< 0.0001
ω-3 PUFA (g), Mean ± SD	1.953 ± 1.434	2.013 ± 1.458	1.851 ± 1.387	0.0008
ALA (g), Mean ± SD	1.776 ± 1.253	1.827 ± 1.249	1.691 ± 1.255	0.0014
SDA (g), Mean ± SD	0.009 ± 0.038	0.009 ± 0.040	0.008 ± 0.033	0.4173
EPA (g), Mean ± SD	0.051 ± 0.177	0.054 ± 0.190	0.045 ± 0.151	0.1276
DPA (g), Mean ± SD	0.021 ± 0.063	0.023 ± 0.070	0.019 ± 0.047	0.1317
DHA (g), Mean ± SD	0.095 ± 0.267	0.100 ± 0.292	0.087 ± 0.218	0.1407
ω-6 PUFA (g), Mean ± SD	17.953 ± 11.512	18.528 ± 11.211	16.981 ± 11.939	< 0.0001
LA (g), Mean ± SD	17.784 ± 11.460	18.350 ± 11.150	16.829 ± 11.906	< 0.0001
AA (g), Mean ± SD	0.169 ± 0.150	0.178 ± 0.161	0.153 ± 0.126	< 0.0001
Race (%)				0.9751
Mexican American	7.77	7.69	7.91	
Non-Hispanic Black	9.17	9.04	9.38	
Non-Hispanic White	74.80	74.94	74.57	
Others	8.26	8.33	8.14	
Education (%)				< 0.0001
Less than high school	16.49	13.11	22.18	
High school	27.14	26.66	27.95	
More than high school	56.37	60.23	49.88	
Hypertension (%)				< 0.0001
No	71.46	80.03	57.01	
Yes	28.54	19.97	42.99	
Diabetes (%)				< 0.0001
No	91.94	96.09	84.93	
Yes	8.06	3.91	15.07	
Cardiovascular disease (%)				< 0.0001
No	88.68	92.66	81.96	
Yes	11.32	7.34	18.04	
Vigorous activity (%)				< 0.0001
No	60.30	52.11	74.10	
Yes	39.70	47.89	25.90	
Moderate activity (%)				< 0.0001
No	43.85	40.18	50.04	
Yes	56.15	59.82	49.96	
Smoking status (%)				< 0.0001
Non smoker	42.71	48.19	33.47	
Previous smoker	29.09	23.72	38.15	
Current smoker	28.20	28.08	28.38	

**Abbreviations**: PUFA, polyunsaturated fatty acid; TPFA, total polyunsaturated fatty acids; BMI, body mass index; AA, arachidonic acid; ALA, α-linolenic acid; DHA, docosahexaenoic acid; LA, linoleic acid; EPA, eicosapentaenoic acid; DPA, docosapentaenoic acid; SDA, octadecanotetraenoic acid; PSM, propensity score matching

After PSM, there were a total of 2280 participants in this study, including 1140 participants with and without ED (Fig. 1). Additional file Table S1 shows their weighted baseline characteristics after PSM. Differences in covariates between the two groups were controlled to some extent (Additional file: Table S1 and Figure S1). Interestingly, AA (*P* < 0.001) remained statistically significant between the two groups.

### Univariate analysis of ED

Additional file Table S2 shows the results of the univariate analysis. All of the covariates included in this study were associated with ED (*P* < 0.05). For PUFAs, TPFA, ω-3 PUFA, ω-6 PUFA, ALA, DPA, LA and AA all reduced the prevalence of ED (OR < 1, *P* < 0.05), especially DPA [OR = 0.26 (0.08, 0.93), *P* = 0.038] and AA [OR = 0.23 (0.14, 0.37), *P* < 0.001].

Covariates such as education, cardiovascular disease, diabetes, and smoking status were associated with ED after PSM (Additional file Table S2). In terms of PUFAs, only AA [OR = 0.35 (0.19, 0.63), *P* < 0.001] maintained robust results as a protective factor for ED.

### The relationships between PUFAs and ED

Table 2 shows the relationship between PUFAs and ED in each of the three adjustment models. TPFA, ω-3 PUFA, ω-6 PUFA, DPA, LA and AA [Model 1: OR = 0.23 (0.14, 0.37); Model 2: OR = 0.29 (0.17, 0.48); Model 3: OR = 0.33 (0.20, 0.56); all *P* < 0.001] all reduced the prevalence of ED under any adjustment model (OR < 1; *P* < 0.05). For ALA, the study showed that ED was related only in model 1 and 2. In addition, for EPA and DHA, ED was found only in model 2 and 3.

**Table 2:** The relationship between PUFA intake and the prevalence of ED before and after PSM

Characteristics	Before PSM			After PSM		
	Model 1 OR (95% CI), *P*	Model 2 OR (95% CI), *P*	Model 3 OR (95% CI), *P*	Model 1 OR (95% CI), *P*	Model 2 OR (95% CI), *P*	Model 3 OR (95% CI), *P*
TPFA	0.98 (0.98, 0.99), **< 0.001**	0.99 (0.98, 1.00), **< 0.001**	0.99 (0.99, 1.00), **0.014**	0.99 (0.99, 1.00), 0.076	0.99 (0.99, 1.00), 0.061	0.99 (0.99, 1.00), 0.091
ω-3 PUFA	0.88 (0.84, 0.92), **< 0.001**	0.92 (0.87, 0.97), **0.001**	0.94 (0.89, 0.99), **0.016**	0.95 (0.90, 1.01), 0.120	0.95 (0.90, 1.01), 0.109	0.95 (0.90, 1.01), 0.125
ALA	0.86 (0.82, 0.91), **< 0.001**	0.92 (0.86, 0.97), **0.004**	0.94 (0.89, 1.00), 0.062	0.96 (0.89, 1.03), 0.224	0.95 (0.89, 1.02), 0.188	0.96 (0.89, 1.03), 0.248
SDA	0.45 (0.08, 2.61), 0.374	0.22 (0.04, 1.41), 0.111	0.20 (0.03, 1.36), 0.099	0.37 (0.04, 3.47), 0.385	0.41 (0.04, 3.90), 0.439	0.31 (0.03, 3.09), 0.319
EPA	0.73 (0.49, 1.09), 0.120	0.65 (0.42, 0.99), **0.046**	0.62 (0.39, 0.97), **0.035**	0.71 (0.42, 1.20), 0.199	0.72 (0.42, 1.22), 0.225	0.67 (0.39, 1.14), 0.142
DPA	0.27 (0.08, 0.93), **0.038**	0.24 (0.07, 0.86), **0.028**	0.24 (0.07, 0.91), **0.035**	0.36 (0.08, 1.53), 0.164	0.40 (0.10, 1.69), 0.214	0.36 (0.08, 1.57), 0.174
DHA	0.80 (0.61, 1.05), 0.102	0.73 (0.55, 0.96), **0.025**	0.74 (0.55, 0.98), **0.038**	0.79 (0.56, 1.10), 0.159	0.80 (0.57, 1.11), 0.184	0.77 (0.55, 1.09), 0.142
ω-6 PUFA	0.98 (0.97, 0.99), **< 0.001**	0.99 (0.98, 1.00), **< 0.001**	0.99 (0.99, 1.00), **0.013**	0.99 (0.99, 1.00), 0.079	0.99 (0.99, 1.00), 0.062	0.99 (0.99, 1.00), 0.094
LA	0.98 (0.97, 0.99), **< 0.001**	0.99 (0.98, 1.00), **< 0.001**	0.99 (0.99, 1.00), **0.015**	0.99 (0.99, 1.00), 0.086	0.99 (0.99, 1.00), 0.068	0.99 (0.99, 1.00), 0.102
AA	0.23 (0.14, 0.37), **< 0.001**	0.29 (0.17, 0.48), **< 0.001**	0.33 (0.20, 0.56), **< 0.001**	0.35 (0.19, 0.63), **< 0.001**	0.34 (0.19, 0.63), **< 0.001**	0.36 (0.20, 0.65), **< 0.001**

**Notes**: Bold fonts indicate *P* < 0.05. Model 1 was a crude model. Model 2 adjusted for age, BMI, race and education. Model 3 added hypertension, cardiovascular disease, diabetes, vigorous activity, moderate activity, and smoking status on the basis of Model 2. **Abbreviations**: PUFA, polyunsaturated fatty acid; TPFA, total polyunsaturated fatty acids; AA, arachidonic acid; ALA, α-linolenic acid; DHA, docosahexaenoic acid; LA, linoleic acid; EPA, eicosapentaenoic acid; DPA, docosapentaenoic acid; SDA, octadecanotetraenoic acid; PSM, propensity score matching; OR, odds ratio; CI, confidence interval

After PSM, regardless of what kind of adjustment model, only AA was linked to a noteworthy decrease in the prevalence of ED [Table 2; Model 1: OR = 0.35 (0.19, 0.63); Model 2: OR = 0.34 (0.19, 0.63); Model 3: OR = 0.36 (0.20, 0.65); all *P* < 0.001].

**Fig. 2:**
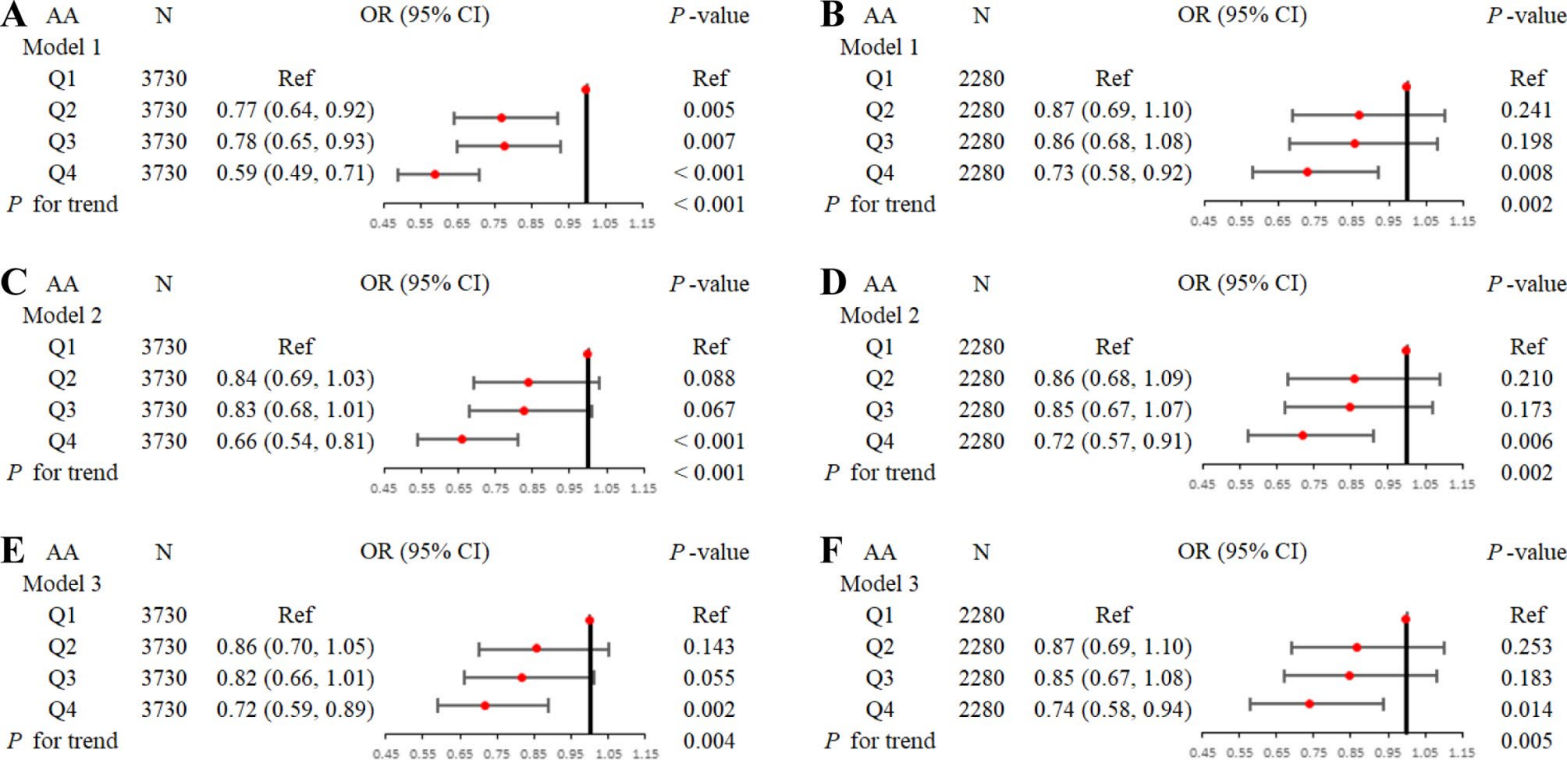
The association between AA intake and the prevalence of ED before and after PSM. **Notes**: Quartile grouping of AA intake: Q1: 0–25%; Q2: 25-50%; Q3: 50-75%; Q4: 75-100%. **A, C, E** stands for before PSM. **B, D, F** stands for after PSM. **Abbreviations**: AA, arachidonic acid; ED, erectile dysfunction; PSM, propensity score matching; OR, odds ratio; CI, confidence interval; Ref, reference

According to the multivariate and univariate analysis results before and after PSM, in this study, the PUFA with the most robust results (i.e., AA) was chosen as the main research object. Figure 2 shows the relationship between AA and ED under the three adjustment models after AA was converted by quaternity (Q1, Q2, Q3 and Q4; Q1 as a reference) before and after PSM. The prevalence of ED was gradually reduced as the AA intake increased under any adjustment model, both before and after PSM [Model 3: Before and after PSM: Q4 vs. Q1: 0.72 (0.59, 0.89) and 0.74 (0.58, 0.94), *P* = 0.002 and 0.014, *P* for trend = 0.004 and 0.005, respectively].

### Evaluation of the relative importance of each variable by the XGBoost algorithm model

**Fig. 3:**
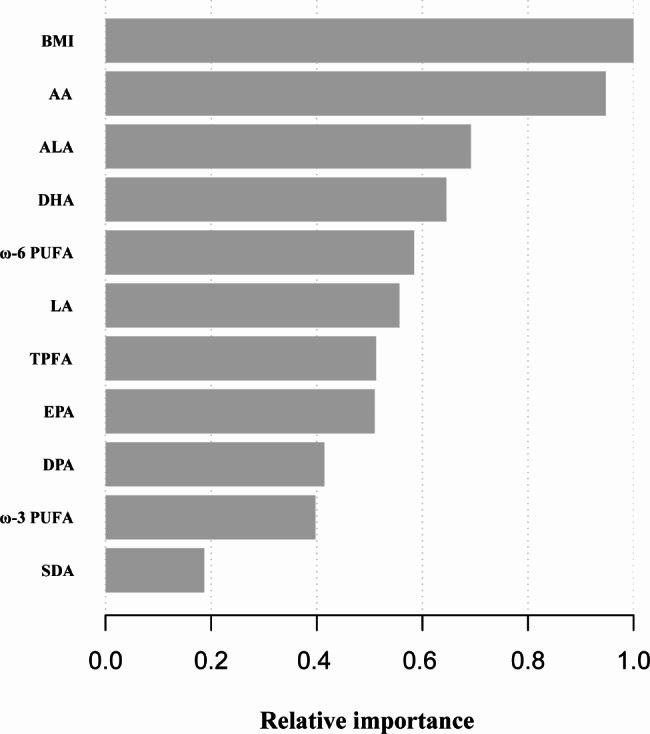
The relative importance of the included variables in the XGBoost algorithm model. **Abbreviations**: PUFA, polyunsaturated fatty acid; TPFA, total polyunsaturated fatty acids; BMI, body mass index; AA, arachidonic acid; ALA, α-linolenic acid; DHA, docosahexaenoic acid; LA, linoleic acid; EPA, eicosapentaenoic acid; DPA, docosapentaenoic acid; SDA, octadecanotetraenoic acid

In this study, by building an XGBoost algorithm model in machine learning, the relative importance of the included variables was predicted. Figure 3 shows that BMI, AA, ALA, DHA and ω-6 PUFA were the five most critical variables. Moreover, the study further found that AA was the most important of all the PUFAs.

### Subgroup analysis between AA and ED

The study was stratified by age, race, education, BMI, hypertension, cardiovascular disease, diabetes, vigorous activity, moderate activity, and smoking status, which were shown in Table 3. AA was associated with a reduced prevalence of ED in people who were aged ≥ 40 years, were non-Hispanic whites; were non smokers; had a high school education or higher and a BMI of 25 to 29.99; had or did not have hypertension, diabetes and engagement in moderate activity; had no cardiovascular disease; and did not engage in vigorous activity (all *P* < 0.05).

**Table 3:** Stratified associations between AA intake and the prevalence of ED before and after PSM

Subgroup	Before PSM			After PSM		
	OR (95% CI)	*P*	*P* for interaction	OR (95% CI)	*P*	*P* for interaction
Age (years)			0.239			0.456
< 40	0.54 (0.21, 1.38)	0.197		0.52 (0.16, 1.71)	0.285	
≥ 40	0.29 (0.15, 0.55)	< 0.001		0.32 (0.15, 0.65)	0.002	
BMI (kg/m^2^)			0.857			0.823
< 25	0.36 (0.13, 1.01)	0.053		0.31 (0.09, 1.05)	0.061	
25-29.99	0.30 (0.13, 0.69)	0.005		0.31 (0.12, 0.81)	0.017	
≥ 30	0.42 (0.16, 1.06)	0.066		0.56 (0.20, 1.57)	0.274	
Race			**0.022**			0.061
Mexican American	1.16 (0.37, 3.66)	0.807		1.61 (0.42, 6.12)	0.483	
Non-Hispanic Black	0.36 (0.13, 1.01)	0.051		0.40 (0.12, 1.37)	0.147	
Non-Hispanic White	0.17 (0.07, 0.37)	< 0.001		0.20 (0.09, 0.49)	< 0.001	
Others	0.85 (0.11, 6.59)	0.879		0.15 (0.01, 2.44)	0.183	
Education			**0.010**			**0.009**
Less than high school	0.88 (0.40, 1.91)	0.741		1.10 (0.46, 2.67)	0.826	
High school	0.18 (0.06, 0.53)	0.002		0.19 (0.06, 0.63)	0.007	
More than high school	0.15 (0.06, 0.37)	< 0.001		0.15 (0.05, 0.42)	< 0.001	
Hypertension			0.429			0.576
No	0.36 (0.19, 0.67)	0.001		0.39 (0.19, 0.78)	0.009	
Yes	0.27 (0.10, 0.72)	0.009		0.26 (0.08, 0.83)	0.022	
Diabetes			0.260			0.312
No	0.37 (0.21, 0.66)	< 0.001		0.40 (0.21, 0.76)	0.006	
Yes	0.19 (0.04, 0.85)	0.030		0.16 (0.02, 1.15)	0.069	
Cardiovascular disease			0.573			0.565
No	0.35 (0.20, 0.60)	< 0.001		0.38 (0.20, 0.72)	0.003	
Yes	0.28 (0.04, 1.85)	0.185		0.21 (0.03, 1.62)	0.133	
Vigorous activity			0.150			0.286
No	0.28 (0.15, 0.53)	< 0.001		0.32 (0.16, 0.65)	0.002	
Yes	0.48 (0.18, 1.33)	0.158		0.64 (0.18, 2.28)	0.494	
Moderate activity			0.478			0.699
No	0.39 (0.20, 0.77)	0.007		0.40 (0.18, 0.89)	0.025	
Yes	0.26 (0.11, 0.59)	0.002		0.30 (0.11, 0.78)	0.014	
Smoking status			0.402			0.518
Non smoker	0.21 (0.09, 0.50)	< 0.001		0.25 (0.09, 0.69)	0.007	
Previous smoker	0.53 (0.20, 1.38)	0.190		0.47 (0.16, 1.38)	0.170	
Current smoker	0.51 (0.19, 1.34)	0.170		0.58 (0.19, 1.82)	0.354	

**Notes**: Bold fonts indicate *P* for interaction < 0.05. All covariates were adjusted in this model without adjusting the stratified variable itself. **Abbreviations**: AA, arachidonic acid; ED, erectile dysfunction; BMI, body mass index; PSM, propensity score matching; OR, odds ratio; CI, confidence interval

The study also revealed that AA and ED had the above characteristics after PSM (Table 3). In addition, AA was found to interact with education both before and after PSM (*P* for interaction = 0.010 and 0.009 before and after PSM, respectively).

### Dose response relationships between AA and ED

**Fig. 4:**
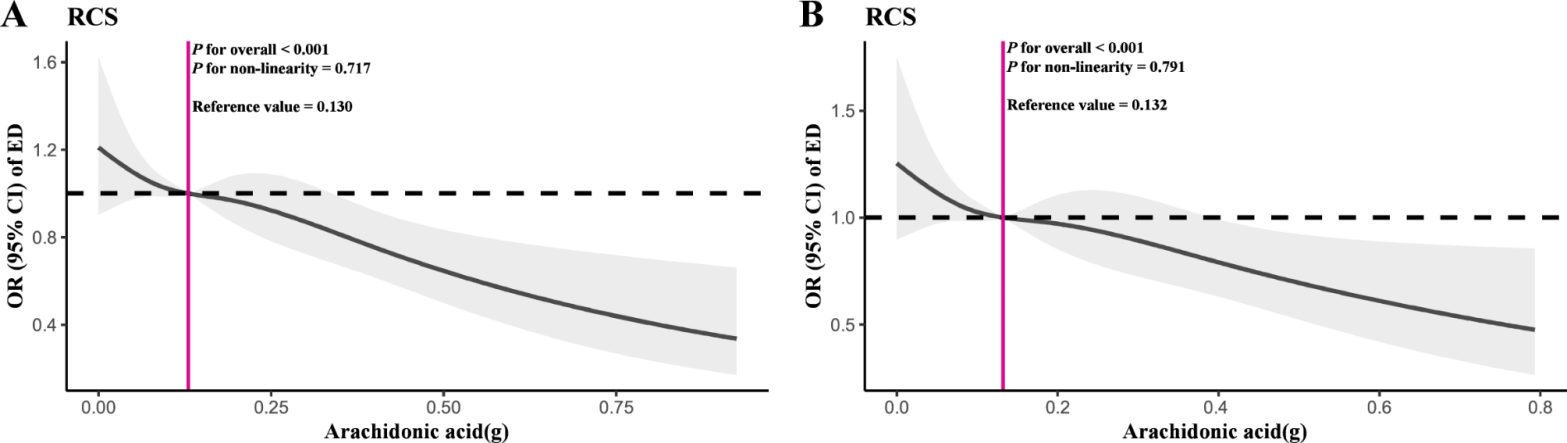
Dose response analysis of AA intake and the prevalence of ED before and after PSM through RCS. **Notes**: **A**: RCS before PSM under the fully adjusted model (Model 3); **B**: RCS after PSM under the fully adjusted model (Model 3). **Abbreviations**: RCS, restricted cubic splines; AA, arachidonic acid; ED, erectile dysfunction; PSM, propensity score matching; OR, odds ratio; CI, confidence interval

The results of the RCS are shown in Fig. 4. Under the fully adjusted model, a nonlinear relationship was not found between AA intake and the prevalence of ED either before or after PSM (Before PSM: *P* for nonlinearity = 0.717; After PSM: *P* for nonlinearity = 0.791). AA intake at OR = 1 was used as a reference value (Reference value before and after PSM = 0.130 and 0.132, respectively), which might be the recommended daily intake of AA for the prevention of ED in people.

**Fig. 5:**
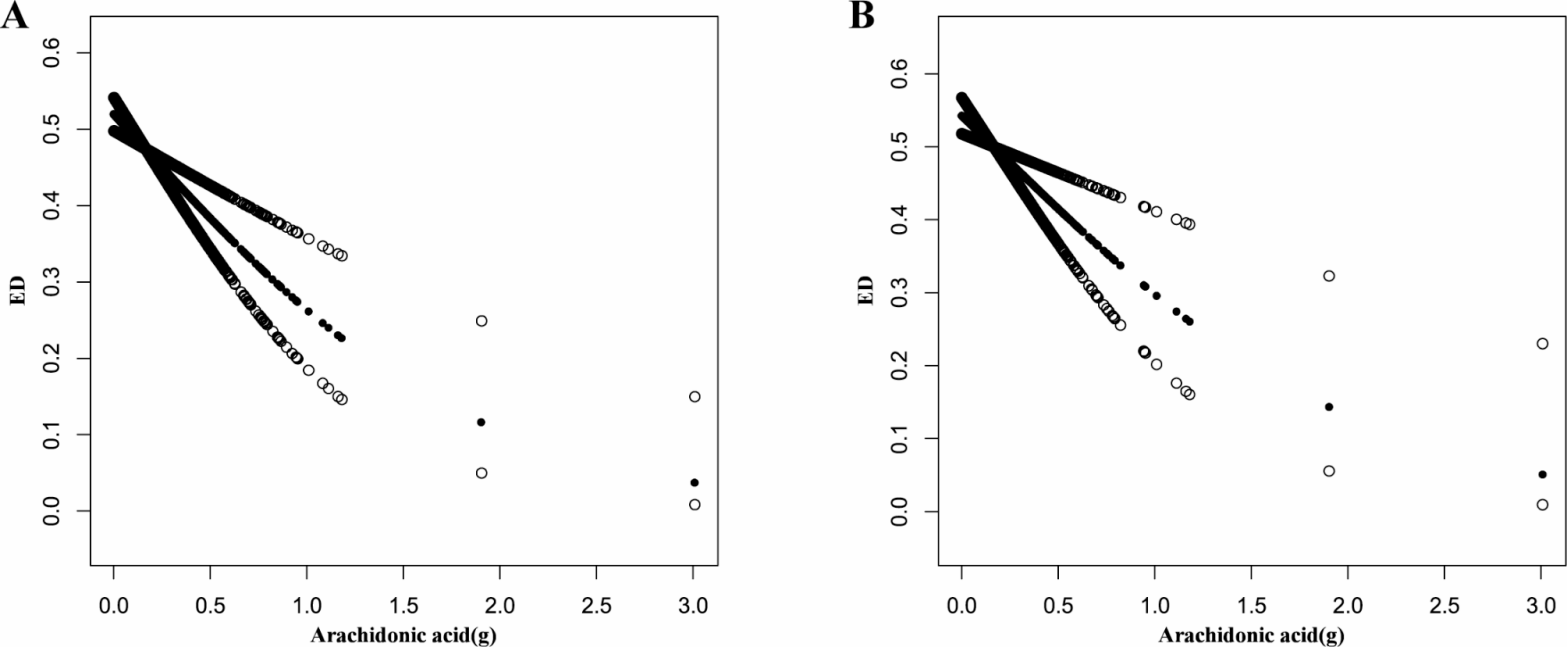
Dose response analysis of AA intake and the prevalence of ED before and after PSM through smooth curve fitting. **Notes**: **A**: Smooth curve fitting before PSM under the fully adjusted model (Model 3); **B**: Smooth curve fitting after PSM under the fully adjusted model (Model 3). **Abbreviations**: AA, arachidonic acid; ED, erectile dysfunction; PSM, propensity score matching; OR, odds ratio; CI, confidence interval

Further, the linear relationship between them was explored through GAM, smooth curve fitting and a threshold effect model based on the RCS results. Figure 5 shows that the prevalence of ED decreased as AA intake increased. In addition, the threshold effect between AA intake and the prevalence of ED is shown in Table 4. No inflection point was observed to be statistically significant (Before and after PSM: log likelihood ratio = 0.341 and 0.645, respectively), so the relationship between them was better explained by a one-line model [Before PSM: OR = 0.33 (0.20, 0.56), *P* < 0.001; After PSM: OR = 0.36 (0.20, 0.65), *P* < 0.001].

**Table 4:** Threshold effect analysis between AA intake and the prevalence of ED before and after PSM

Outcome: ED	OR (95% CI), *P*	
	Before PSM	After PSM
**Model 1**		
Linear effect	0.33 (0.20, 0.56), < 0.001	0.36 (0.20, 0.65), < 0.001
**Model 2**		
Inflection point (K)	0.331	0.109
AA < K	0.44 (0.20, 0.96), 0.038	0.19 (0.01, 2.90) 0.233
AA > K	0.19 (0.05, 0.72), 0.014	0.40 (0.19, 0.85) 0.016
Log likelihood ratio	0.341	0.645

**Note**: All covariates were adjusted in this model. **Abbreviations**: AA, arachidonic acid; ED, erectile dysfunction; PSM, propensity score matching; OR, odds ratio; CI, confidence interval

### Exploration of potential mechanistic links

**Fig. 6:**
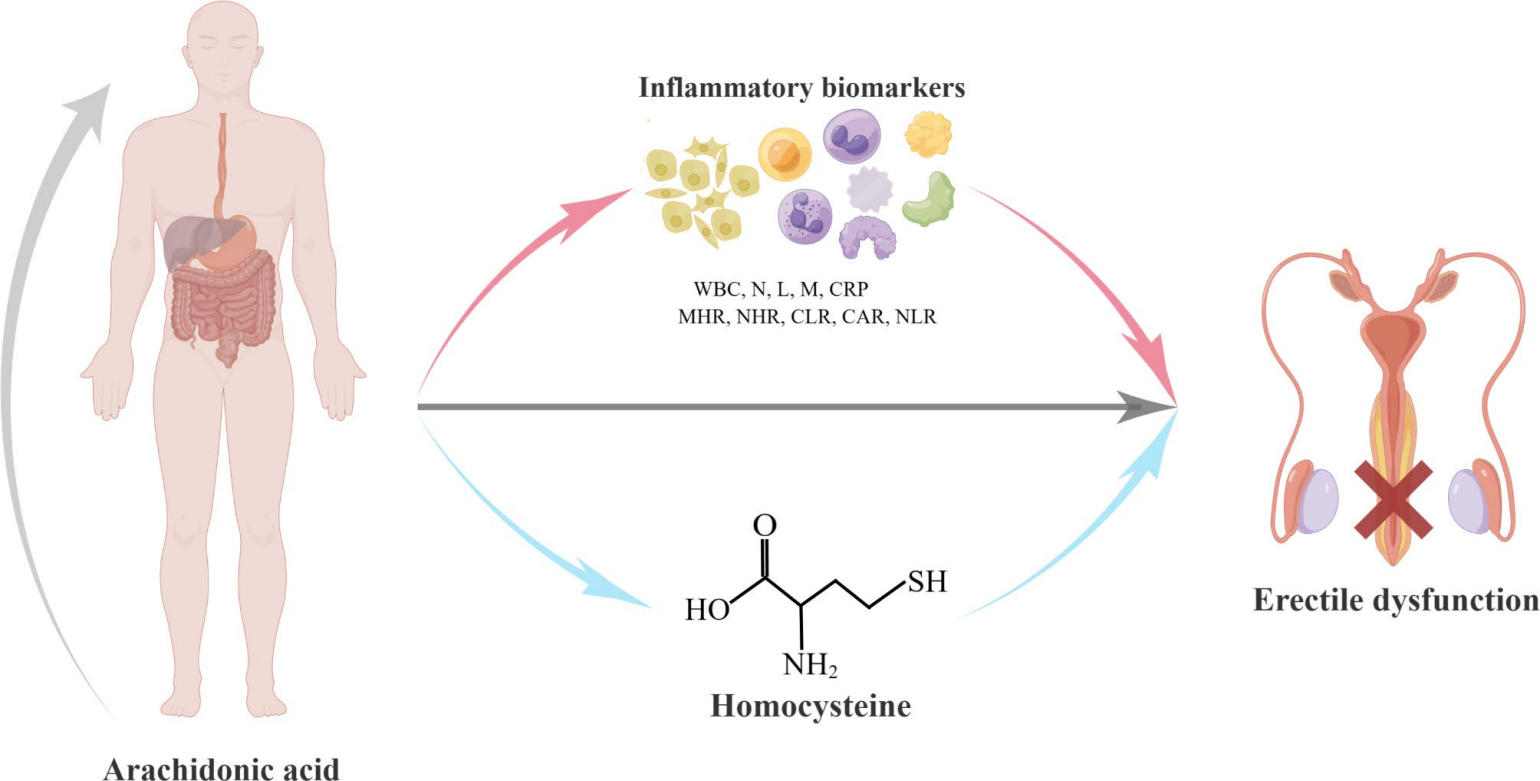
Intuitive diagram of AA and ED analysis. **Abbreviations**: AA, arachidonic acid; ED, erectile dysfunction; WBC, white blood cell; N, neutrophils; L, lymphocytes; M, monocytes; CRP, C-reactive protein; MHR, monocyte-to-HDL cholesterol ratio; NHR, neutrophil-to-HDL cholesterol ratio; CLR, CRP-to-lymphocyte ratio; CAR, CRP-to-albumin ratio; NLR, neutrophil-to-lymphocyte ratio

An intuitive diagram is shown in Fig. 6, which was produced by Figdraw. Under the fully adjusted model, the study evaluated the association of AA with inflammatory biomarkers and homocysteine. As shown in Table 5, the results showed that AA had a statistically significant association with MHR [β = -0.062 (-0.112, -0.013), *P* = 0.014], NHR [β = -0.427 (-0.806, -0.047), *P* = 0.028], NLR [β = -0.319 (-0.576, -0.061), *P* = 0.015] and homocysteine [β = -1.327 (-2.255, -0.399), *P* = 0.005].

**Table 5:** The association between AA and inflammatory biomarkers and homocysteine

Mediating factors	N	β (95% CI)	*P*-value
WBC (1000 cells/μl)	3683	-0.313 (-0.911, 0.286)	0.306
N (1000 cells/μl)	3683	-0.254 (-0.592, 0.083)	0.140
L (1000 cells/μl)	3683	-0.010 (-0.438, 0.419)	0.965
M (1000 cells/μl)	3683	-0.037 (-0.081, 0.008)	0.104
CRP (mg/l)	3683	-1.547 (-3.502, 0.408)	0.121
MHR	3683	-0.062 (-0.112, -0.013)	**0.014**
NHR	3683	-0.427 (-0.806, -0.047)	**0.028**
CLR	3683	-1.224 (-2.753, 0.306)	0.117
CAR	3683	-0.039 (-0.090, 0.012)	0.135
NLR	3683	-0.319 (-0.576, -0.061)	**0.015**
Homocysteine (μmol/l)	3722	-1.327 (-2.255, -0.399)	**0.005**

**Notes**: Bold fonts indicate *P* < 0.05. All covariates were adjusted in this model. **Abbreviations**: AA, arachidonic acid; WBC, white blood cell; N, neutrophils; L, lymphocytes; M, monocytes; CRP, C-reactive protein; MHR, monocyte-to-HDL cholesterol ratio; NHR, neutrophil-to-HDL cholesterol ratio; CLR, CRP-to-lymphocyte ratio; CAR, CRP-to-albumin ratio; NLR, neutrophil-to-lymphocyte ratio; CI, confidence interval

## Discussion

This is the first article to explore the association between the two factors on a large sample size. Based on univariate analysis and multivariate regression analysis results before and after PSM, this study chose AA as the main research object. The highest relative importance of AA among all PUFAs was also shown in the XGBoost algorithm model. Regardless of the adjustment model, increased AA intake reduced the prevalence of ED. RCS, GAM and smooth curve fitting all supported a linear negative association between AA intake and the prevalence of ED. In addition, by exploring the underlying mechanism, the study found that arachidonic acid intake was associated with a decrease in inflammatory biomarkers and homocysteine. This indicates that AA may reduce the prevalence of ED by exerting anti-inflammatory and anti-endothelial injury effects to some extent.

ED is a disease with a multifactorial pathogenesis. A prospective cross-sectional study showed higher levels of inflammation and oxidative stress in the ED group [11]. A study conducted in dialysis patients showed that low levels of systemic inflammation was significantly associated with the severity of ED [33]. Hydrogen, as a novel antioxidant, has been shown to improve ED [37]. Smoking, metabolic syndrome, overweight or obesity, alcohol consumption, and lack of physical activity have been identified as modifiable risk factors for ED, and interestingly, these factors are all strongly associated with proinflammatory states [38]. Therefore, a significant role is played by inflammation and oxidative stress in the occurrence and development of ED.

Under oxidative stress and chronic inflammation, the endothelium is damaged, its function is compromised, and endothelial integrity is a key component of penile erection [9, 39]. Under sexual stimulation, nitric oxide (NO) and other endothelial factors are released by vascular endothelial cells, which block the venous return of erectile tissue, relax the arterial smooth muscle of erectile tissue and then the penis blood flow is increases. Eventually, blood is trapped in the cavernosa, and pressure in the cavity increases significantly, leading to an erection [9]. With endothelial dysfunction, the production and release of physiological NO are hindered, and the contraction and dilation of related blood vessels are impaired [40]. Specifically, there is a lack of available endothelial NO synthase (eNOs), decreased reactivity to NO, increased reactivity to vasoconstriction, and changes in structure, such as thickening, increased permeability, macrophage deposition etc. [41, 42]. In addition, tumor necrosis factor-α (TNF-α) produced in response to inflammation and oxidative stress not only inhibits the expression of eNOs but also induces reactive oxygen species (ROS) production, which may directly reduce NO levels [30, 40].

AA is one of the main dietary long-chain ω-6 PUFAs, dairy products, eggs, fish, and meat are the main sources [43]. The mechanism by which increased AA intake reduces the prevalence of ED is unclear, but this study suggests that it may be the results of the following: First, studies have found that increasing AA intake to 1000–1500 mg/d did not affect inflammatory markers [44]. Roberts et al. found that supplementation with AA reduced inflammatory responses to training in resistance-trained males [45]. Roy et al. found that supplementation with AA effectively alleviated obesity-induced inflammation in mice [46]. Second, Nakano et al. found that dietary supplements of AA improved age-related endothelial dysfunction [47]. In this study, the majority of the population was ≥ 40 years old (60.24%), and AA was hypothesized to reduce the prevalence of ED by protecting endothelial function. Homocysteine is an important substance that induces endothelial injury [48]. The results shown in Table 5 confirm the conjecture of this study to some extent. Furthermore, a study by Mariam et al. showed that epoxyeicosatrienoic acid (EET), a cytochrome P450 metabolite of AA, induced cavernosomal relaxation in mice through activation of the cGMP/NO pathway, ATP-sensitive K^+^ channels and BK channels [49]. Previous studies have shown that EET is produced in endothelial cells of the rat penis and that EET antagonists impair erectile function [50]. These results suggest that metabolites of AA may have potential preventive and therapeutic effects on ED. Finally, testosterone therapy could be used to treat ED patients with low testosterone levels [2]. Pablo et al. showed that exogenous AA induced testosterone secretion through the lipoxygenase pathway [51]. The underlying mechanisms need to be further elucidated in future studies.

### Study strengths and limitations

Several advantages can be found in the present research. First, the study had a large sample size and used a stricter classification of ED, which increases the credibility of the results. Second, in this study, AA was selected as the main research object through univariate analysis and multivariate regression analysis results before and after PSM. Then, the relative importance of AA among all PUFAs is proven by the XGBoost algorithm model in machine learning. Third, through a stratified analysis before and after PSM, the study identified covariates interacting with AA. In addition, the RCS, GAM, threshold effect analysis, and smooth curve fitting results indicated a linear negative correlation between AA intake and the prevalence of ED that can be explained by a single-line model. Finally, the exploration of the underlying mechanism suggests that AA intake and reduced prevalence of ED may be related to anti-inflammatory and anti-endothelial injury.

Limitations can also be found in this study. First, the study population was American; the eating habits and eating patterns different in different countries, which limits the regional generalizability of the study results. Second, as 2001–2002 data collected only once dietary recall data, resulting in the inapplicability of the method of averaging dietary intake. Third, the study categorized ED by questionnaires, which could have led to certain memory biases. In addition, owing to the limited information contained in the database, the study did not include testosterone levels and the specific types of ED could not be categorized, which made it impossible to evaluate specific types of ED. Finally, due to the cross-sectional study design in NHANES, causal inference cannot be made, and consequently the exploration of potential mechanisms can only be regarded as hypothesis generation, which needs to be explored through further mechanistic research and longitudinal research.

## Conclusion

The results show that AA intake is linear negatively correlated with the prevalence of ED. In addition, the potential mechanistic links between AA and ED may be related to anti-inflammatory and anti-endothelial injury effects to some extent. This study aimed to raise awareness about dietary intervention in disease development. The prevention of ED through the intake of arachidonic acid also provides a new approach by which clinicians can manage patients with ED.

### Electronic supplementary material

Below is the link to the electronic supplementary material.

**Additional file 1: Figure S1**. The balance of the data set before and after PSM. **Table S1**. The weighted basic characteristics of the study population after PSM. **Table S2**. Univariate analysis of the relationship between variables and ED before and after PSM

## Data Availability

Publicly available database was utilized in this study. All the details are available on the website (https://www.cdc.gov/nchs/nhanes).

## List of abbreviations

AA: Arachidonic acid
ALA: α-linolenic acid
BMI: Body mass index
CAR: CRP-to-albumin ratio
CI: Confidence interval
CLR: CRP-to-lymphocyte ratio
CRP: C-reactive protein
DHA: Docosahexaenoic acid
DPA: Docosapentaenoic acid
ED: Erectile dysfunction
EET: Epoxyeicosatrienoic acid
eNOs: Endothelial NO synthase
EPA: Eicosapentaenoic acid
GAM: Generalized additive model
L: Lymphocytes
LA: Linoleic acid
LTs: Leukotrienes
M: Monocytes
MHR: Monocyte-to-HDL cholesterol ratio
N: Neutrophils
NCHS: National Center for Health Statistics
NHANES: National Health and Nutrition Examination Survey
NHR: Neutrophil-to-HDL cholesterol ratio
NLR: Neutrophil-to-lymphocyte ratio
NO: Nitric oxide
OR: Odds ratio
PGs: Prostaglandins
PSM: Propensity score matching
PUFAs: Polyunsaturated fatty acids
RCS: Restricted cubic splines
Ref: Reference
ROS: Reactive oxygen species
SDA: Octadecanotetraenoic acid
TNF-α: Tumor necrosis factor-α
TPFA: Total polyunsaturated fatty acids
TXs: Thromboxanes
VIF: Variance inflation factor
WBC: White blood cell
