# Supplementary material for: Association between polyunsaturated fatty acid intake and the prevalence of erectile dysfunction: A cross-sectional analysis of the NHANES 2001–2004
Source: Lipids Health Dis. 2023 Oct 25;22:182. doi: 10.1186/s12944-023-01950-9 (PMC10601238; doi:10.1186/s12944-023-01950-9)
Supplement: Supplementary file 1 — Additional file 1: Figure S1. The balance of the data set before and after PSM. Table S1. The weighted basic characteristics of the study population after PSM. Table S2. Univariate analysis of the relationship between variables and ED before and after PSM [file 12944_2023_1950_MOESM1_ESM.docx]

**Figure S1.** The balance of the data set before and after PSM.


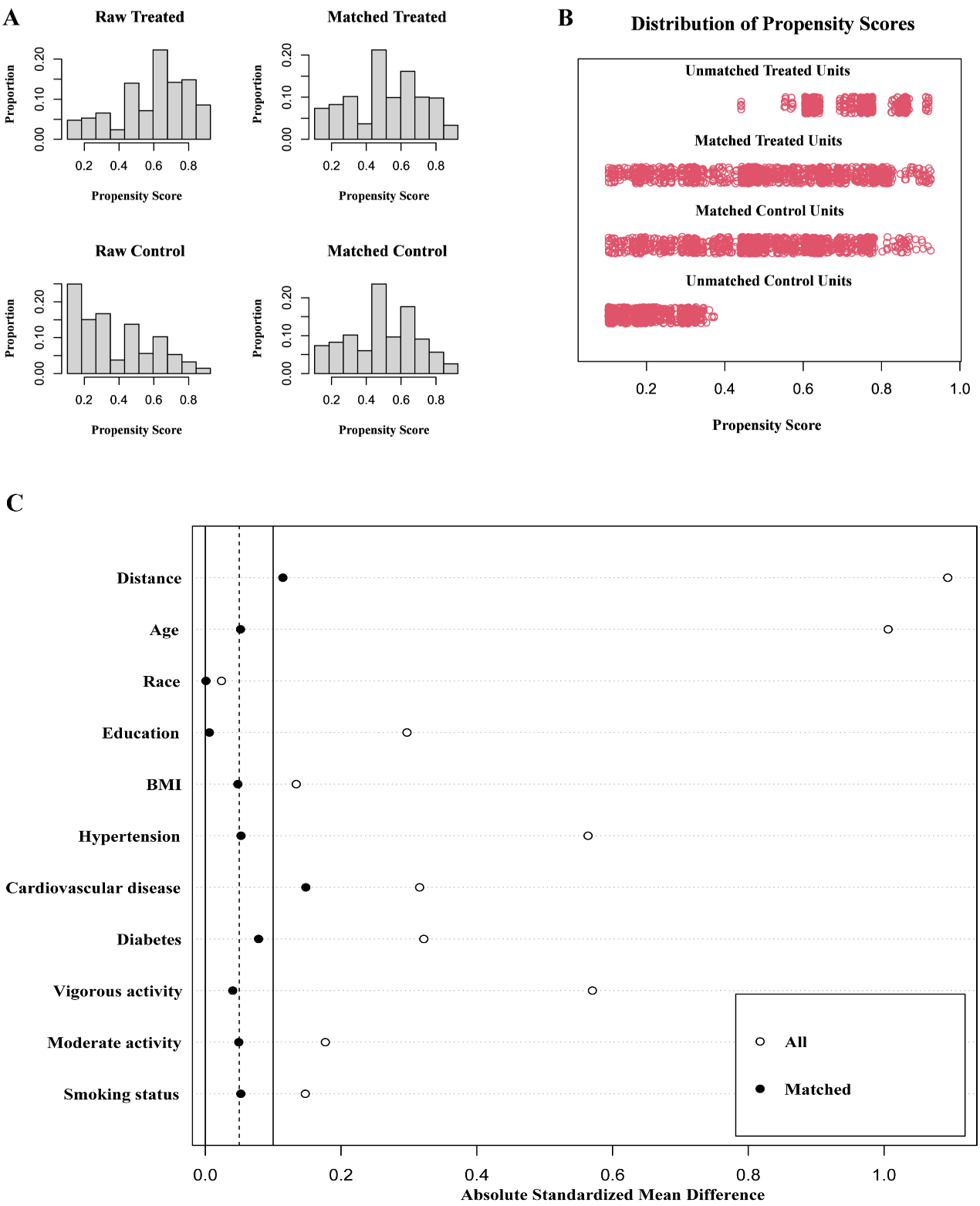


**Notes:** A was the histogram of propensity score distribution of the raw data and the matched data; B was the jitter graph of propensity score distribution of raw data and matched data; C was the Love plot of absolute standardized mean difference (SMD) of all covariates in raw data and matched data. **Abbreviations:** BMI, Body mass index.

**Table S1.** The weighted basic characteristics of the study population after PSM.

| **Characteristics** | **Total** | **Erectile dysfunction** | | ***P*-value** |
| --- | --- | --- | --- | --- |
|  |  | **No** | **Yes** |  |
| Total participants | 2280 | 1140 | 1140 | - |
| Age (%) |  |  |  | 0.3632 |
| < 40 years | 26.31 | 25.58 | 27.27 |  |
| ≥ 40 years | 73.69 | 74.42 | 72.73 |  |
| BMI (kg/m^2^), Mean ± SD | 28.40 ± 5.43 | 28.28 ± 5.10 | 28.54 ± 5.85 | 0.2634 |
| TPFA (g), Mean ± SD | 20.025 ± 12.489 | 20.349 ± 11.896 | 19.597 ± 13.221 | 0.1546 |
| ω-3 PUFA (g), Mean ± SD | 1.940 ± 1.440 | 1.985 ± 1.484 | 1.880 ± 1.378 | 0.0851 |
| ALA (g), Mean ± SD | 1.767 ± 1.249 | 1.796 ± 1.237 | 1.730 ± 1.263 | 0.2108 |
| SDA (g), Mean ± SD | 0.008 ± 0.038 | 0.009 ± 0.044 | 0.007 ± 0.028 | 0.1983 |
| EPA (g), Mean ± SD | 0.049 ± 0.166 | 0.054 ± 0.187 | 0.042 ± 0.132 | 0.0948 |
| DPA (g), Mean ± SD | 0.021 ± 0.067 | 0.023 ± 0.081 | 0.019 ± 0.043 | 0.2018 |
| DHA (g), Mean ± SD | 0.094 ± 0.272 | 0.104 ± 0.323 | 0.082 ± 0.183 | 0.0627 |
| ω-6 PUFA (g), Mean ± SD | 17.743 ± 11.182 | 18.033 ± 10.545 | 17.359 ± 11.962 | 0.1543 |
| LA (g), Mean ± SD | 17.575 ± 11.129 | 17.855 ± 10.480 | 17.203 ± 11.925 | 0.1665 |
| AA (g), Mean ± SD | 0.168 ± 0.151 | 0.178 ± 0.165 | 0.155 ± 0.128 | 0.0005 |
| Race (%) |  |  |  | 0.4264 |
| Mexican American | 7.88 | 7.14 | 8.86 |  |
| Non-Hispanic Black | 8.44 | 8.15 | 8.81 |  |
| Non-Hispanic White | 75.56 | 76.54 | 74.27 |  |
| Others | 8.12 | 8.16 | 8.06 |  |
| Education (%) |  |  |  | 0.2806 |
| Less than high school | 15.02 | 15.44 | 14.45 |  |
| High school | 32.45 | 31.10 | 34.23 |  |
| More than high school | 52.54 | 53.46 | 51.32 |  |
| Hypertension (%) |  |  |  | 0.3632 |
| No | 69.99 | 69.23 | 70.99 |  |
| Yes | 30.01 | 30.77 | 29.01 |  |
| Diabetes (%) |  |  |  | 0.0111 |
| No | 92.18 | 93.42 | 90.54 |  |
| Yes | 7.82 | 6.58 | 9.46 |  |
| Cardiovascular disease (%) | |  |  | 0.0199 |
| No | 88.44 | 89.80 | 86.65 |  |
| Yes | 11.56 | 10.20 | 13.35 |  |
| Vigorous activity (%) | |  |  | 0.8148 |
| No | 67.56 | 67.36 | 67.82 |  |
| Yes | 32.44 | 32.64 | 32.18 |  |
| Moderate activity (%) | |  |  | 0.0088 |
| No | 46.73 | 44.36 | 49.88 |  |
| Yes | 53.27 | 55.64 | 50.12 |  |
| Smoking status (%) |  |  |  | 0.0062 |
| Non smoker | 38.24 | 40.98 | 34.61 |  |
| Previous smoker | 31.90 | 29.94 | 34.50 |  |
| Current smoker | 29.86 | 29.08 | 30.89 |  |

**Abbreviations:** PUFA, polyunsaturated fatty acid; TPFA, total polyunsaturated fatty acids; BMI, body mass index; AA, arachidonic acid; ALA, α-linolenic acid; DHA, docosahexaenoic acid; LA, linoleic acid; EPA, eicosapentaenoic acid; DPA, docosapentaenoic acid; SDA, octadecanotetraenoic acid; PSM, propensity score matching; SD, standard deviation.

**Table S2.** Univariate analysis of the relationship between variables and ED before and after PSM.

| **Characteristics** | **Before PSM** | | **After PSM** | |
| --- | --- | --- | --- | --- |
|  | **Statistics** | **Erectile dysfunction** | **Statistics** | **Erectile dysfunction** |
|  |  | **OR (95% CI), *P*** |  | **OR (95% CI), *P*** |
| Age (%) |  |  |  |  |
| < 40 years | 39.76 | 1 | 26.31 | 1 |
| ≥ 40 years | 60.24 | 5.88 (5.02, 6.89), < 0.001 | 73.69 | 1.11 (0.91, 1.35), 0.300 |
| BMI (kg/m^2^), Mean ± SD | 28.08 ± 5.44 | 1.03 (1.01, 1.04), < 0.001 | 28.40 ± 5.43 | 1.01 (0.99, 1.03), 0.234 |
| TPFA (g), Mean ± SD | 20.257 ± 12.840 | 0.98 (0.98, 0.99), < 0.001 | 20.025 ± 12.489 | 0.99 (0.99, 1.00), 0.076 |
| ω-3 PUFA (g), Mean ± SD | 1.953 ± 1.434 | 0.88 (0.84, 0.92), < 0.001 | 1.940 ± 1.440 | 0.95 (0.90, 1.01), 0.120 |
| ALA (g), Mean ± SD | 1.776 ± 1.253 | 0.86 (0.81, 0.91), < 0.001 | 1.767 ± 1.249 | 0.96 (0.89, 1.03), 0.224 |
| SDA (g), Mean ± SD | 0.009 ± 0.038 | 0.45 (0.08, 2.61), 0.374 | 0.008 ± 0.038 | 0.37 (0.04, 3.47), 0.385 |
| EPA (g), Mean ± SD | 0.051 ± 0.177 | 0.73 (0.49, 1.09), 0.120 | 0.049 ± 0.166 | 0.71 (0.42, 1.20), 0.199 |
| DPA (g), Mean ± SD | 0.021 ± 0.063 | 0.26 (0.08, 0.93), 0.038 | 0.021 ± 0.067 | 0.36 (0.08, 1.53), 0.164 |
| DHA (g), Mean ± SD | 0.095 ± 0.267 | 0.80 (0.61, 1.04), 0.102 | 0.094 ± 0.272 | 0.79 (0.56, 1.10), 0.159 |
| ω-6 PUFA (g), Mean ± SD | 17.953 ± 11.512 | 0.98 (0.97, 0.99), < 0.001 | 17.743 ± 11.182 | 0.99 (0.99, 1.00), 0.079 |
| LA (g), Mean ± SD | 17.784 ± 11.460 | 0.98 (0.97, 0.99), < 0.001 | 17.575 ± 11.129 | 0.99 (0.99, 1.00), 0.086 |
| AA (g), Mean ± SD | 0.169 ± 0.150 | 0.23 (0.14, 0.37), < 0.001 | 0.168 ± 0.151 | 0.35 (0.19, 0.63), < 0.001 |
| Race (%) |  |  |  |  |
| Mexican American | 7.77 | 1 | 7.88 | 1 |
| Non-Hispanic Black | 9.17 | 0.83 (0.67, 1.02), 0.078 | 8.44 | 0.91 (0.69, 1.19), 0.482 |
| Non-Hispanic White | 74.80 | 0.97 (0.82, 1.15), 0.751 | 75.56 | 1.03 (0.84, 1.28), 0.752 |
| Others | 8.26 | 0.74 (0.55, 0.99), 0.044 | 8.12 | 0.84 (0.57, 1.22), 0.354 |
| Education (%) |  |  |  |  |
| Less than high school | 16.49 | 1 | 15.02 | 1 |
| High school | 27.14 | 0.59 (0.49, 0.71), < 0.001 | 32.45 | 1.35 (1.08, 1.69), 0.008 |
| More than high school | 56.37 | 0.47 (0.40, 0.55), < 0.001 | 52.54 | 1.07 (0.87, 1.31), 0.531 |
| Hypertension (%) |  |  |  |  |
| No | 71.46 | 1 | 69.99 | 1 |
| Yes | 28.54 | 3.58 (3.11, 4.12), < 0.001 | 30.01 | 1.12 (0.95, 1.33), 0.187 |
| Diabetes (%) |  |  |  |  |
| No | 91.94 | 1 | 92.18 | 1 |
| Yes | 8.06 | 3.82 (3.03, 4.83), < 0.001 | 7.82 | 1.39 (1.06, 1.83), 0.018 |
| Cardiovascular disease (%) |  |  |  |  |
| No | 88.68 | 1 | 88.44 | 1 |
| Yes | 11.32 | 3.09 (2.52, 3.78), < 0.001 | 11.56 | 1.68 (1.31, 2.15), < 0.001 |
| Vigorous activity (%) |  |  |  |  |
| No | 60.30 | 1 | 67.56 | 1 |
| Yes | 39.70 | 0.34 (0.29, 0.39), < 0.001 | 32.44 | 0.92 (0.77, 1.10), 0.385 |
| Moderate activity (%) |  |  |  |  |
| No | 43.85 | 1 | 46.73 | 1 |
| Yes | 56.15 | 0.70 (0.62, 0.80), < 0.001 | 53.27 | 0.91 (0.77, 1.07), 0.240 |
| Smoking status (%) |  |  |  |  |
| Non smoker | 42.71 | 1 | 38.24 | 1 |
| Previous smoker | 29.09 | 2.67 (2.28, 3.12), < 0.001 | 31.90 | 1.47 (1.21, 1.79), < 0.001 |
| Current smoker | 28.20 | 1.26 (1.07, 1.49), 0.005 | 29.86 | 1.10 (0.90, 1.35), 0.343 |

**Abbreviations:** PUFA, polyunsaturated fatty acid; TPFA, total polyunsaturated fatty acids; BMI, body mass index; AA, arachidonic acid; ALA, α-linolenic acid; DHA, docosahexaenoic acid; LA, linoleic acid; EPA, eicosapentaenoic acid; DPA, docosapentaenoic acid; SDA, octadecanotetraenoic acid; PSM, propensity score matching; SD, standard deviation; OR, odds ratio; CI, confindence interval.
